# Associations between Coparenting Relationships and Maternal Depressive Symptoms and Negative Bonding to Infant

**DOI:** 10.3390/healthcare9040375

**Published:** 2021-03-28

**Authors:** Yoko Takeishi, Yasuka Nakamura, Mikako Yoshida, Maiko Kawajiri, Fumi Atogami, Toyoko Yoshizawa

**Affiliations:** 1 Department of Women’s Health Nursing & Midwifery, Tohoku University Graduate School of Medicine, Miyagi 9808575, Japan; nakamurayasuka@nursing.med.tohoku.ac.jp (Y.N.); mikako.yoshida.e2@tohoku.ac.jp (M.Y.); mkawajiri@med.tohoku.ac.jp (M.K.); toyoko@nursing.med.tohoku.ac.jp (T.Y.); 2 Department of Nursing, Kurume University, Fukuoka 8300011, Japan; atogami_fumi@med.kurume-u.ac.jp

**Keywords:** attachment, childcare, coparenting, couple relationship, mental health

## Abstract

Maternal mental illnesses during early postpartum may be caused by lack of the coparenting relationship parents share and cooperate regarding child-rearing. This study clarifies the association of the coparenting relationship and negative mental health of mothers at one and three months after childbirth. This study conducted a secondary analysis of data from an intervention study wherein 24 mothers rearing their first child with a cohabitant (husband/partner) participated. Maternal mental health was evaluated using the Edinburgh Postnatal Depression Scale to determine postpartum depressive symptoms and the Mother-to-Infant Bonding Scale to assess negative bonding. Mothers’ average age was 31.5 ± 4.2 years old. All mothers were not working during the research period. The prevalence of postpartum depression and bonding disorder were approximately 13% and 21%, respectively. A better coparenting relationship was associated with lower postpartum depressive symptoms at both one month (*β* = −0.617, *p* = 0.002) and three months (*β* = −0.709, *p* < 0.01) postpartum. In contrast, no association was found between a coparenting relationship and negative bonding. The results indicate that the coparenting relationship may possibly prevent maternal depression during the early postpartum period.

## 1. Introduction

Postpartum depression, caused by unfamiliarity with child-rearing, is a mental illness suffered by some mothers [1,2,3,4,5]; approximately 10% of mothers experience depressive symptoms within one postpartum year [5]. Peak prevalence of postpartum depression, 13%, has been found to occur in the first three to four months postpartum [6]. Postpartum depression is sometimes characterized by the mother not feeling affection toward her infant. Bonding disorder, a lack of emotional linkage between mothers and infants, is one of the mental illness and becomes negative in the context of absent affection, hate, neglect, and impulses to harm [7,8,9]. These bonding disorder symptoms presented more frequently within the first three to four months after childbirth [8,9]. Researchers have speculated that these two negative mental health states are related to new mothers experiencing and accumulating psychological stress day by day, associated with unfamiliarity with child-rearing, immediately after birth [10]. Thus, it is imperative that mothers receive support to prevent depressive symptoms and facilitate bonding with their infant. In the context of Japan, a postpartum care project recently initiated by the government has begun to identify and treat women with postpartum depression within one month after giving birth; however, the incidence of postpartum depression has not yet decreased [5].

Regarding other aspects of etiology for mental illnesses during child-rearing, the parents’ child-rearing relationship may affect mothers [2,3,4,11]. In Japan, mothers generally have a major child-rearing role; mothers who have preschoolers usually spend approximately 4.5 hours a day on childcare and housework, whereas fathers spend approximately one hour [12]. Because of this, mothers feel burdened physically and psychologically. This is further aggravated, as they do not receive appreciation from the fathers [2,11]. Furthermore, most fathers only care for their infant at night and on their days-off because they work on other family and work responsibilities during the day [13]. Mothers, however, demand more efforts in childrearing from the fathers without regard for the latter’s fatigue due to daytime work. Thus, fathers become stressed and lose motivation to engage in childrearing [11]. These differences in perception of the other’s situation and a lack of appreciative behavior, and the need to adjust to the other’s situation, may induce depressive symptoms and negative bonding.

The parents’ relationship (regarding childrearing) is vital in resolving maternal mental illnesses. Feinberg (2003) and Van Egeren (2004) introduced a concept called “coparenting”, which embodies a new framework for the parents’ relationship [11,12]. It differs from the traditional concept wherein ideal childrearing requires quantitatively equal contributions from the mothers and fathers. In contrast, coparenting involves agreement on the childrearing roles dependent on individual physical ability and specialty (e.g., breastfeeding), practice and cooperation in labor, sharing of economic and social responsibilities for child development and growth, and respect for the other’s efforts. Previous studies have found an association between the coparenting relationship and mental illness, indicating that better coparenting could lead to a decrease in depressive symptoms and impulses to harm the child among women six to 13 months postpartum [13,14,15]. However, this association between coparenting and mental health is still unclear for women in the early postpartum period. Therefore, this study examined the association between coparenting and mental health specifically regarding depressive symptoms and negative bonding at one and three months postpartum among mothers.

## 2. Materials and Methods 

### 2.1. Study Design and Participants

This study was a secondary analysis of data collected in a quasi-experimental study conducted in Japan between July 2016 and May 2017 [16]. During pregnancy, the quasi-experimental study provided mothers with an educational program; 17 mothers received the education program as the intervention group, while the other seven were part of the control group. No significant differences regarding demographics were found between the groups [16], so the data of all 24 mothers were combined and then analyzed in the current study.

First-time mothers who met the following criteria were included for analysis: (1) married or living with partner/husband and (2) 20 years old and above. The exclusion criteria were existence of a psychiatric disease and multiple pregnancies.

Mothers were recruited through flyers, direct explanation, or chain-referral-sampling. One of the researchers placed flyers on a bulletin board and explained the study directly to pregnant women during childbirth classes conducted in obstetrics/gynecology clinics and hospitals. Mothers accessed an application form and read the study explanation documents. Subsequently, they sent their consent forms by mail. After enrollment, mothers answered a questionnaire to collect sociodemographic data either by mail or an online system. Mothers also answered anonymous questionnaires regarding depressive symptoms and bonding to the infant at one and three months after birth. The Ethics Committee of Tohoku University Graduate School of Medicine approved this study (No. 2016-1-326).

### 2.2. Measurements

Maternal mental health (including depressive symptoms and negative bonding) was assessed using scales at one and three months after childbirth. The childrearing status, socio-demographic, and obstetrical data were also collected:

#### 2.2.1. Depressive Symptoms

The Edinburgh Postnatal Depression Scale (EPDS) [17] was used to assess postpartum depression, including three items that assessed negative mood, related to unadjusted childrearing practice. A higher score in the scale indicated a higher risk of experiencing depressive symptoms. The cut-off with a Japanese sample is 8/9 point of the total score [18]. In the current study, Cronbach’s alpha was 0.88.

#### 2.2.2. Negative Bonding to Infant

The Mother-to-Infant Bonding Scale (MIBS) was used to evaluate bonding between mother and infant [8]. Ten-item in the MIBS include absence of affection, hate, rejection, or impulse to harm one’s own infant. A higher score in the scale indicated a higher risk of being bonding disorder. The cut-off has not been sure yet among Japanese women because item number is different from the other countries’ one [19]. The Cronbach’s alpha was 0.65 for the current study.

#### 2.2.3. Coparenting Relationship

The coparenting relationship was measured by the Coparenting Relationship Scale (CRS) [20]. CRS uses a seven-point Likert scale that consists of seven subscales, namely, Agreement, Closeness, Exposure to conflict, Support, Undermining, Endorse partner’s parenting, and Division of labor. A higher score indicated a better coparenting relationship, meaning that as a couple, the mother and her partner/husband worked together and cooperated effectively regarding their child-rearing. In this study, Cronbach’s alpha was 0.72.

#### 2.2.4. Childrearing Status

This status included actual childrearing practices and social support. Actual child-rearing practice was assessed with original questions regarding father’s childrearing time during workdays and days-off. In Japan, most mothers do not return to work for over six months after childbirth [21], which was the case for mothers in this study, as well. They also spend most of their day on childcare. Given this, the mothers’ childrearing time was not collected. Instead, the manner of feeding their infant was queried and whether it was breastfeeding or formula milk or another. A question on sources of social support was also asked and whether they received support from relatives (e.g., mother’s parents or others, such as the father’s parents and other social resources), or no help at all.

#### 2.2.5. Sociodemographic and Obstetrical Data

Socio-demographic data, including maternal age, educational level (nine to 12 years or more), and current occupational status (employed or unemployed), were obtained at 36 weeks of gestation when participants were recruited. The marital relationship was also measured at recruitment by the Marital Love Scale (MLS), which evaluates marital quality in terms of romance, affection, and love [22]. The score ranged from 10 to 70. A higher score indicated better marital quality. In this study, Cronbach’s alpha was 0.91.

Regarding obstetrical data, information on perceived planning for pregnancy (Planned (whenever)/Unplanned), number of prenatal education classes attended, infertility treatment (yes/no), and pregnancy complications (yes/no) was collected at 36 weeks of gestation. Additionally, information on the gestational age at childbirth and type of childbirth (vaginal birth or caesarean section) was obtained at one month after childbirth.

### 2.3. Statistical Analysis

Analysis was performed using IBM SPSS version 25.0 for Windows (SPSS Inc., Chicago, IL, USA). The significance level was set at less than 0.05.

Univariate analysis was performed using Pearson’s correlation coefficient to evaluate the relationship between mental health and the continuous variables, and the Student’s *t*-test was used to compare mental health by the categorical variables. Subsequently, multiple regression analysis with forced entry was conducted to reveal the association mental health with independent variables. The independent variables were selected by variables as less than 0.1 of *p*-value in univariate analysis, or theoretical importance. Notably, multicollinearity was confirmed before conducting multiple regression analysis.

## 3. Results

Out of 24 mothers, the average age was 31.5 ± 4.2 years old, employees were 11 (45.8%), the current pregnancy of 10 (41.7%) were unplanned, and 20 (83.3%) experienced vaginal birth (Table 1). Except 1 mother giving birth in 36 weeks of gestation, all mothers had term delivery.

The average EPDS score was 5.7 ± 4.0 and 4.6 ± 5.2 at one and three months, respectively. Three mothers (12.5%) scored more than nine points (maximum 15 points) on the EPDS at one month postpartum and maintained a score of more than nine points (maximum 19 points) at three months. The average MIBS score was 2.3 ± 2.3 and 1.3 ± 1.6 at one and three months, respectively.

EPDS scores at both one month (*r* = −0.560, *p* = 0.004) and three months (*r* = −0.864, *p* < 0.001) were negatively correlated with CRS score for the same time points (Table 2). At one month, the EPDS score tended to be higher among mothers with higher educational levels (*t* = 1.846, *p* = 0.078). At three months, the EPDS score tended to be higher among mothers who received child-rearing support from their own parents (*t* = −1.863, *p* = 0.076). The MIBS score at each time point did not correlate with CRS score or the other variables.

The multiple regression model (Table 3) showed the EPDS score at one month postpartum was significantly associated with CRS score (*β* = −0.617, *p* = 0.002) and education level (*β* = −0.397, *p* = 0.029). The EPDS score at three months postpartum was also significantly associated with CRS score (*β* = −0.709, *p* < 0.01), while association with education level disappeared. The EPDS score at three months postpartum tended to associate with father’s child-rearing time in day-off (*β* = −0.184, *p* = 0.092). The MIBS score at each time point was not significantly associated with any variables.

## 4. Discussion

This study revealed that three mothers who scored more than nine points on the EPDS were at risk of postpartum depression. In addition, we found that a better coparenting relationship was associated with lower EPDS score at one and three months postpartum. In contrast, no association was found between coparenting and negative bonding.

Even though the current study used a small sample, the prevalence of postpartum depression was approximately 13%. This is consistent with the results of a previous systematic review [6], which reported a peak prevalence of postpartum depression of 12.9% at three months postpartum. This systematic review of 28 studies also found this mental illness appeared starting in the early postpartum period [6]. Considering these results, mothers are at risk of mental illness in the early postpartum period.

In this study, the better coparenting relationship significantly decreased the EPDS score. It can be speculated that agreement on mothers’ and fathers’ childrearing roles is vital in preventing maternal depression [12]. To arrive at such agreement, couples must understand the other’s social situation, and individual physical ability and specialty for childrearing. This understanding may dissuade mothers from demanding more childrearing roles from the fathers [23], and instead, appreciate the father’s efforts in other areas [24]. This would result in less frustration experienced by the mothers towards the fathers. With this, it is clear that agreement on the childrearing roles, based on the parents understanding each other, is important for first time couples to prevent postpartum depression.

Education level was associated with lower EPDS scores at one month postpartum, which is consistent with a previous cohort study conducted in Japan [25], which pointed out that education level reflected socioeconomic status. A higher education level meant higher economic status [25], which was related to being employed and having the ability to find social support easily, which can prevent postpartum depression [2,4].

Additionally, the current study found that fathers’ expanded child-rearing time on days off may prevent maternal depression at three months but not at one month postpartum. The median of fathers’ child-rearing time on days off was 270 min at three months postpartum (data not shown), which was slightly more than that at one month postpartum (240 min). This increase in fathers’ child-rearing time was assumed to be due to changing child-rearing needs from one month to three months postpartum. At one month, child-rearing often involves much time spent breastfeeding, which can only be done by the mother. As the child grows, fathers become able to participate in child-rearing, for example playing with their infant at three months postpartum. This shift in responsibility from mother to father could let mothers take a temporary break (e.g., nap, leisure etc.) from child-rearing, subsequently allowing them to recover physically and psychologically [26].

The coparenting relationship was associated with EPDS score but not with MIBS score (i.e., negative bonding). Postpartum depression occurs when child-rearing demands overwhelm the mother’s ability to cope with psychological stress that related child-rearing [3,4,10]. An association between coparenting and postpartum depressive symptoms was observed in the current study, which supports the idea that support and agreement in the coparenting relationship can reduce child-rearing demands for one parent (mother/father). On the other hand, bonding is a psychological linkage between the infant and one parent. The degree of this linkage depends on the parent’s interpersonal relationship with his/her parents that has been cultivated as he/she (current parent) grows [27,28]. Negative bonding behavior, such as harm to the child or neglect, is assumed to occur when psychological distance between the parent and his/her infant is closer than comfortable interpersonal relationship for the parent. This bond might not be affected by the independent child-rearing-related relationship with a partner, which could be why an association between the coparenting relationship and negative bonding was not observed.

Regarding generalization of our study results, for mothers having more than two children, the coparenting relationship must change because child-rearing labor and its must evolve based on how the children are growing up. The result of this study regarding how better coparenting involves understanding each other and agreement on the childrearing roles would also be applicable to mothers with more than two children, even though their coparenting relationship has already developed since the first childbirth. Adjusting the coparenting relationship, such as agreement on the direction of childrearing, cooperation in increased labor, and appreciation to one another, would be the next steps for them.

This study has several limitations. First, information was not collected on treatment for depression and bonding disorder. Such information may affect the results. However, most participants did not have any mental health disorder. As it takes a few months to recover the state of one’s mental health disorder after beginning treatment, most participants did not seem to be receiving treatment. Second, participants were limited to married mothers cohabiting her partner/husband. As such, the results of the study cannot be adopted for unmarried mothers, regardless of whether or not they live with her partner/husband. Because unmarried mothers are known to be at high risk, health care providers already take care of them from pregnancy. Thus, the focus of this study was placed on low-risk mothers who generally are not attended to by health care providers. Third, this is a secondary analysis of interventional study data. There may be a bias in that participants had relatively better coparenting relationships than the rest of the Japanese population during childrearing. Further research is needed to confirm the association between the coparenting relationship and mental health, which can be done through a larger sample of low-risk parents.

## 5. Conclusions

This study clarified that approximately 13% of participating mothers were at risk of postpartum depression and bonding disorder, respectively, in the early postpartum period. This study also showed a better coparenting relationship was associated with lower postpartum depressive symptoms at both one and three months postpartum. In contrast, no association was found between coparenting and negative bonding. Further study is needed to be able to generalize the associations shown in this study to a large population.

## Figures and Tables

**Table 1:** Socio-demographic and obstetrical characteristics (*n* = 24).

Socio-Demographic and Obstetrical Variables	*n* (%)
Age (years)	31.5 ± 4.2
Educational level	
9–12 years	8 (33.3)
>12 years	16 (66.7)
Occupational status	
Employed	11 (45.8)
Unemployed	13 (54.2)
Marital relationship (MLS)	40.6 ± 10.0
Perceived planning for pregnancy	
Planned/Whenever	14 (58.3)
Unplanned	10 (41.7)
Participation in prenatal education	2.0 ± 1.8
Infertility treatment (yes)	7 (29.2)
Pregnancy complications (yes)	7 (29.2)
Gestational age of childbirth (weeks)	39.0 ± 1.3
Types of childbirth	
Vaginal birth	20 (83.3)
Caesarean section	4 (16.7)

MLS: Marital Love Scale. Data are *n* (%) or mean ± standard deviation. Occupational status was obtained at 36 weeks of gestation.

**Table 2:** Comparison of childrearing and parental mental health at one and three months after birth.

Variables	EPDS 1 Month		EPDS 3 Months		MIBS 1 Month		MIBS 3 Months	
	M ± SD or *r*	*p*	M ± SD or *r*	*p*	M ± SD or *r*	*p*	M ± SD or *r*	*p*
Scores	5.7 ± 4.0	-	4.6 ± 5.2	-	2.3 ± 2.3	-	1.3 ± 1.6	-
Time of recruitment								
Age	0.019	0.929	0.064	0.766	0.098	0.648	0.159	0.458
Educational level		0.078		0.124		0.545		0.796
9–12 years	7.8 ±4.3		7.8 ± 7.6		2.8 ± 2.2		1.4 ± 1.8	
>12 years	4.7 ± 3.6		3.0 ± 2.4		2.1 ± 2.4		1.2 ± 1.6	
Marital relationship (MLS)	0.113	0.598	−0.110	0.610	0.011	0.958	−0.194	0.363
Perceived planning for pregnancy		0.914		0.382		0.686		0.260
Planned/Whenever	5.8 ± 3.5		3.8 ± 4.6		2.5 ± 2.5		0.9 ± 1.3	
Unplanned	5.6 ± 4.8		5.7 ± 5.9		2.1 ± 2.2		1.7 ± 2.0	
Participation in prenatal education	0.201	0.346	0.166	0.438	−0.050	0.817	0.217	0.307
Infertility treatment		0.662		0.872		0.802		0.639
Yes	6.3 ± 4.6		4.9 ± 6.5		2.1 ± 2.2		1.0 ± 1.5	
No	5.5 ± 3.9		4.5 ± 4.7		2.4 ± 2.4		1.4 ± 1.7	
Pregnancy complications		0.669		0.795		0.227		0.947
Yes	5.1 ± 3.7		4.1 ± 3.7		1.4 ± 1.8		1.3 ± 1.5	
No	5.9 ± 4.2		4.8 ± 5.7		2.7 ± 2.4		1.2 ± 1.7	
Childbirth								
Gestational age at childbirth	0.047	0.826	−0.009	0.966	0.149	0.487	0.026	0.905
Types of childbirth		0.523		0.559		0.316		0.512
Vaginal birth	6.0 ± 4.3		4.3 ± 4.6		2.6 ± 2.6		1.4 ± 1.7	
Caesarean section	4.5 ± 2.5		6.0 ± 8.3		1.3 ± 1.9		0.8 ± 1.0	
First month postpartum								
Coparenting relationship								
CRS Total	−0.560	0.004			−0.119	0.579		
Coparenting Agreement	−0.548	0.006			−0.243	0.253		
Coparenting Closeness	−0.383	0.064			−0.231	0.277		
Exposure to Conflict	−0.563	0.004			0.064	0.765		
Coparenting Support	−0.222	0.298			0.138	0.519		
Coparenting Undermining	−0.553	0.005			−0.321	0.126		
Endorsement of Partner’s Parenting	−0.338	0.106			−0.032	0.881		
Division of Labor	−0.364	0.080			−0.009	0.965		
Feeding of baby		0.118				1.000		
Breastfeeding only	4.4 ± 3.2				2.3 ± 2.6			
Using formula	7.0 ± 4.5				2.3 ± 2.1			
Support for child-rearing		0.676				0.939		
Mother’s parents	5.6 ± 4.2				2.4 ± 2.4			
Others/nobody	6.5 ± 3.7				2.3 ± 2.1			
Father’s child-rearing time								
Work day	00.197				−0.235			
Day off	−0.107				−0.182			
Third month postpartum								
Coparenting relationship								
CRS Total			−0.864	<0.001			−0.154	0.474
Coparenting Agreement			−0.783	<0.001			−0.237	0.265
Coparenting Closeness			−0.672	<0.001			0.043	0.843
Exposure to Conflict			−0.756	<0.001			−0.138	0.521
Coparenting Support			−0.672	<0.001			0.002	0.992
Coparenting Undermining			−0.583	0.003			−0.289	0.170
Endorsement of Partner’s Parenting			−0.761	<0.001			−0.187	0.383
Division of Labor			−0.668	<0.001			−0.082	0.455
Feeding of baby				0.353				0.188
Breastfeeding only			3.9 ± 4.6				0.9 ±1.4	
Using formula			6.0 ± 6.3				1.9 ± 2.0	
Support for child-rearing				0.099				0.639
Mother’s parents			2.9 ± 2.6				1.4 ± 1.7	
Others/nobody			8.6 ± 7.6				1.0 ± 1.5	
Father’s child-rearing time								
Work day			−0.224	0.253			−0.235	0.405
Day off			−0.320	0.105			−0.182	0.302

EPDS: Edinburgh Postnatal Depression Scale; MIBS: Mother-to-Infant Bonding Scale; MLS: Marital Love Scale; CRS: Coparenting Relationship Scale. Pearson’s correlation coefficient (*r*) or Student’s *t*-test (mean ± standard deviation) was conducted depending on continuous or categorical variables, respectively.

**Table 3:** Factors associated with mental health at one month and three months after childbirth.

Independent Variables	EPDS 1 Month		EPDS 3 Months		MIBS 1 Month		MIBS 3 Months	
	*β*	*p*	*β*	*p*	*β*	*p*	*β*	*p*
CRS Total	−0.617	0.002	−0.709	<0.001	−0.132	0.585	−0.246	0.383
Father’s child-rearing time on days off	−0.034	0.844	−0.184	0.092	−0.161	0.486	−0.112	0.625
Support for childrearing	−0.169	0.361	0.154	0.202	−0.056	0.822	−0.215	0.407
Educational level	−0.397	0.029	−0.124	0.276	−0.132	0.567	0.025	0.917
Adjusted R^2^	0.370		0.763		0.063		0.075	

EPDS: Edinburgh Postnatal Depression Scale; MIBS: Mother-to-Infant Bonding Scale; CRS: Coparenting Relationship Scale. Support for child-rearing: support from mother’s parent = 0, support from others or no support = 1. Educational level: 9–12 years = 0, >12 years = 1. Multiple regression analysis was conducted for each EPDS and MIBS score, and the independent variables corresponding to the same time points were used.

## Data Availability

The data presented in this study are available on request from the corresponding author. The data are not publicly available due to privacy restrictions.

## References

[1] Shorey S., Chee C.Y.I., Ng E.D., Chan Y.H., Tam W.W.S., Chong Y.S. (2018). Prevalence and incidence of postpartum depression among healthy mothers: A systematic review and meta-analysis. J. Psychiatr. Res..

[2] O’hara M.W., Swain A.M. (1996). Rates and risk of postpartum depression—A meta-analysis. Int. Rev. Psychiatry.

[3] Beck C.T. (1996). A meta-analysis of predictors of postpartum depression. Nurs. Res..

[4] Beck C.T. (2001). Predictors of postpartum depression: An update. Nurs. Res..

[5] Takehara K., Suto M., Kato T. (2020). Parental psychological distress in the postnatal period in Japan: A population-based analysis of a national cross-sectional survey. Sci. Rep..

[6] Gavin N.I., Gaynes B.N., Lohr K.N., Meltzer-Brody S., Gartlehner G., Swinson T. (2005). Perinatal depression: A systematic review of prevalence and incidence. Obstet. Gynecol..

[7] Kumar R.C. (1997). “Anybody’s child”: Severe disorders of mother-to-infant bonding. Br. J. Psychiatry.

[8] Yoshida K., Yamashita H., Conroy S., Marks M., Kumar C. (2012). A Japanese version of mother-to-infant bonding scale: Factor structure, longitudinal changes and links with maternal mood during the early postnatal period in Japanese mothers. Arch. Womens Ment. Health.

[9] Brockington I. (2004). Postpartum psychiatric disorders. Lancet.

[10] Skipstein A., Janson H., Kjeldsen A., Nilsen W., Mathiesen K.S. (2012). Trajectories of maternal symptoms of depression and anxiety over 13 years: The influence of stress, social support, and maternal temperament. BMC Public Health.

[11] Feinberg M.E. (2003). The internal structure and ecological context of coparenting: A framework for research and intervention. Parent Sci. Pract..

[12] Van Egeren L.A., Hawkins D.P. (2004). Coming to terms with coparenting: Implications of definition and measurement. J. Adult Dev..

[13] Feinberg M.E., Kan M.L. (2008). Establishing family foundations: Intervention effects on coparenting, parent/infant well-being, and parent-child relations. J. Fam. Psychol..

[14] Feinberg M.E., Jones D.E., Hostetler M.L., Roettger M.E., Paul I.M., Ehrenthal D.B. (2016). Couple-focused prevention at the transition to parenthood, a randomized trial: Effects on coparenting, parenting, family violence, and parent and child adjustment. Prev. Sci..

[15] Solmeyer A.R., Feinberg M.E. (2011). Mother and father adjustment during early parenthood: The roles of infant temperament and coparenting relationship quality. Infant Behav. Dev..

[16] Takeishi Y., Nakamura Y., Kawajiri M., Atogami F., Yoshizawa T. (2019). Developing a prenatal couple education program focusing on coparenting for Japanese couples: A quasi-experimental study. Tohoku J. Exp. Med..

[17] Cox J. (1996). Validation of the Edinburgh Postnatal Depression Scale (EPDS) in non-postnatal women. J. Affect. Disord..

[18] Yamashita H., Yoshida K., Nakano H., Tashiro N. (2000). Postnatal depression in Japanese women: Detecting the early onset of postnatal depression by closely monitoring the postpartum mood. J. Affect. Disord..

[19] Bienfait M., Maury M., Haquet A., Faillie J.-L., Franc N., Combes C., Daudé H., Picaud J.-C., Rideau A., Cambonie G. (2011). Pertinence of the self-report Mother-to-Infant Bonding Scale in the neonatal unit of a maternity ward. Early Hum. Dev..

[20] Feinberg M.E., Brown L.D., Kan M.L. (2012). A multi-domain self-report measure of coparenting. Parenting.

[21] Kaneko A., Kaneita Y., Yokoyama E., Miyake T., Harano S., Suzuki K., Ibuka E., Tsutsui T., Yamamoto Y., Ohida T. (2006). Factors associated with exclusive breast-feeding in Japan: For activities to support child-rearing with breast-feeding. J. Epidemiol..

[22] Kawashima A., Ito K., Sugawara M., Sakai A., Sugawara K., Kitamura T. (2008). Characteristics of the relationship attribution measure (RAM) with Japanese middle-aged couples. Jpn. J. Psychol..

[23] Biehle S.N., Mickelson K.D. (2012). First-time parents’ expectations about the division of childcare and play. J. Fam. Psychol..

[24] Buckley C.K., Schoppe-Sullivan S.J. (2010). Father involvement and coparenting behavior: Parents’ nontraditional beliefs and family earner status as moderators. Pers. Relatsh..

[25] Matsumura K., Hamazaki K., Tsuchida A., Kasamatsu H., Inadera H., Japan Environment and Children’s Study (JECS) Group (2019). Education level and risk of postpartum depression: Results from the Japan Environment and Children’s Study (JECS). BMC Psychiatry.

[26] Richter D., Krämer M.D., Tang N.K.Y., Montgomery-Downs H.E., Lemola S. (2019). Long-term effects of pregnancy and childbirth on sleep satisfaction and duration of first-time and experienced mothers and fathers. Sleep.

[27] Hall R.A.S., Hoffenkamp H.N., Tooten A., Braeken J., Vingerhoets A.J.J.M., Van Bakel H.J.A. (2015). Child-rearing history and emotional bonding in parents of preterm and full-term infants. J. Child Fam. Stud..

[28] Kitamura T., Ohashi Y., Murakami M., Goto Y. (2014). Anger and perceived parenting: A study of a Japanese population. Psychol. Behav. Sci..

